# Sleep disturbances are associated with increased pain, disease activity, depression, and anxiety in ankylosing spondylitis: a case-control study

**DOI:** 10.1186/ar4054

**Published:** 2012-10-11

**Authors:** Yan Li, Shengli Zhang, Jian Zhu, Xuna Du, Feng Huang

**Affiliations:** 1 Department of Rheumatology, Chinese PLA General Hospital, 28 Fuxing Road, Beijing 100853, China; 2 Department of Child and Adolescent Health, College of Public Health, Zhengzhou University, 100 Kexue Road, Zhengzhou 450001, China; 3 Department of Rheumatology, Fuzhou General Hospital of Nanjing Military Command, 156 Xi'erhuan North Road, Fuzhou 350025, China

## Abstract

**Introduction:**

Literature data suggest that sleep disturbances are prevalent among patients with ankylosing spondylitis (AS) and have a close correlation with pain. Other studies indicate that sleep disturbances are constantly accompanied by depression and anxiety in AS, but their interrelations are poorly understood. This study was designed to evaluate sleep disturbances and their association with demographic variables, pain, disease-specific variables, functional status, covering depression and anxiety in AS patients.

**Methods:**

The 314 patients with AS and age- and sex-matched controls took part in the study, completed a battery of questionnaires, and participated in long-term follow-up. Blood samples were taken to measure C-reactive protein (CRP) and the erythrocyte sedimentation rate (ESR). The association among sleep, pain, disease activity, functional status, depression, and anxiety were assessed by using Pearson/Spearman correlations and multiple regression analysis.

**Results:**

The Pittsburgh Sleep Quality Index (PSQI) score of the Chinese version was significantly higher in the AS group than in the control group (*P *= 0.020). Of the 314 patients with AS, 184 (58.6%) had a high risk for sleep disturbances. The PSQI score was associated with age, years of education, ESR, CRP, overall assessment of health, pain, morning stiffness, Bath Ankylosing Spondylitis Disease Activity Index (BASDAI), Bath Ankylosing Spondylitis Functional Index (BASFI), depression, and anxiety (all *P *< 0.001), but were not associated with disease duration, fingertip-to-floor distance, and Bath Ankylosing Spondylitis Metrology Index (BASMI) (*P *> 0.05). In hierarchic multiple regression analysis, the medical and psychological variables contributed significantly to the variance in sleep-disturbances scores, adding an additional 23.9% to the overall *R^2 ^*beyond that accounted for by demographic variables (R-square, 8.5%), resulting in a final *R^2^*of 42.6%. Multiple stepwise regression analysis revealed that anxiety was the maximal statistical contribution in predicting sleep disturbances (standardized coefficients, 0.287).

**Conclusions:**

The prevalence of sleep disturbances in AS patients is higher than it is generally thought to be. Depression, anxiety, nocturnal pain, and total back pain are the major contributors of sleep disturbances in AS.

## Introduction

Ankylosing spondylitis (AS) is a chronic inflammatory disease that affects approximately 0.36% of the Chinese population [1]. It has a negative effect on all aspects of a patient's life: physically, psychologically, and socially. Sleep disturbances are often reported in rheumatic diseases and are considered to be multifactorial. Some studies document poor quality of sleep in rheumatoid arthritis (RA), osteoarthritis (OA), systemic lupus erythematosus (SLE), fibromyalgia, Sjögren's syndrome, and spondyloarthritis (SpA) [2-7]. Currently, the pathophysiology of sleep disturbances is ill defined, and it is likely to be underreported by AS patients. One possible reason for the lack of therapeutic success in sleep disturbances is the very limited research conducted on the frequency and correlates of sleep disturbances in patients with AS.

Sleep disturbances reduce an individual's quality of life by seriously impairing cognition, mood, and physical symptoms of diseases. A cross-sectional study investigating the effects of sleep deprivation on cognition and work performance of residents and interns in Korea reported severe sleep deprivation (average night sleep, less than 4 hours) was associated with a higher level of stress, more-frequent attention deficit, and difficulty in learning [8]. In a very large sample of 5,877 participants aged between 15 and 54 years, reduced quantity of sleep seemed to be associated with difficulties in coping with stressful life events [9]. Sleep also plays an important role in learning processes and memory consolidation; poor sleep usually is associated with decreased academic performance and reduced neurobehavioral functioning [10]. Moreover, a number of longitudinal studies [11-13] indicate that insomnia or poor sleep is a risk factor for major depression and anxiety. Presumably, effects of poor sleep on cognition and mood are due to reducing cerebral blood flow and metabolic rate in the thalamus, prefrontal and parietal cortices, and elevating the activity of the stress systems (the autonomic sympathoadrenal system and the hypothalamic-pituitary-adrenal system) [14,15]. Furthermore, sleep alterations increase the pathologic significance of any disease and reduce general well-being [16,17]. Clinical studies reported that sleep problems are inversely associated with the pain threshold at all sites, suggesting a defect in central pain processing [2]. Martin [18] suggested a cause-and-effect relation between sleep dysfunction, psychological effects, and musculoskeletal function changes; the greatest effect of sleep disturbances would be a reduction in high-intensity physical exercise tolerance. Therefore, poor sleep could cause a negative impact on exercise and rehabilitation of AS patients. Few studies have been conducted to explore the relation among sleep and physical and psychological symptoms.

Although, the prevalence, type, and severity of sleep complaints in an AS population have been difficult to judge, it has been reported to occur in 15.4% to 80% of patients with AS, reflecting divergent definitions of sleep disturbance and instruments of measure. Hultgren *et al*. [19] studied 43 male and 27 female patients with AS, completed a sleep questionnaire, and the results were compared with earlier findings in 3,558 persons randomly selected from the general population. Too little sleep was reported by 80.8% of the female and 50.0% of the male patients, compared with 28.8% and 21.8%, respectively, in the reference group. Another study [7] assessed the sleep quality of patients with spondyloarthropathy (SpA) with the PSQI in Canada; 69% of the sample were classified as poor sleepers, and worse functional status was associated with poorer sleep quality, longer sleep latency, shorter sleep duration, and poorer sleep efficiency. A recent study [20] of sleep disturbance, assessed by the fourth item of the Hamilton anxiety scale, was found in 15.4% of 110 patients with AS. Although sleep complaints are commonly reported in rheumatism, little is known in persons with AS. Moreover, it is unclear whether the severity of AS is directly related to poor sleep quality. Specifically, given the considerable adverse effects of sleep disturbances on clinical outcomes, identifying the factors associated with poor sleep is an important goal. Studies addressing this issue have had some limitations: small sample size, inclusion of patients with SpA, and lack of comprehensive assessment of sleep quality and physical and psychological status.

The objective of this study were (1) to determine the prevalence of sleep disturbances and their association with demographic variables, disease-specific variables, and other variables that can have an impact on sleep; and (2) to evaluate the statistical contribution in predicting sleep disturbances in AS patients.

## Methods

For long-term follow-up, patients were recruited from two rheumatology clinics and departments in the Chinese PLA General Hospital, and the healthy controls were recruited from three communities. All patients met the 1984 modified New York classification criteria for AS [21]. The 102 age- and sex-matched healthy controls (84 men, 18 women) were included in this case-control study. Controls were group matched by age groups (15 to 20 years, 21 to 25 years, 26 to 30 years, and 30 years and older), meaning that the proportion of controls in each age group was identical to the proportion of patients in each age group. The patients and controls who had malignancy, fibromyalgia, serious infections or systemic diseases, and other chronic diseases were excluded from the study. The study was approved by the Chinese PLA General Hospital ethics committee, and all participants gave their written informed consent, according to the Declaration of Helsinki.

Demographic characteristics, age, years of education, occupation, marital status, disease duration, and duration of morning stiffness were documented for each patient. The patients were subjected to a detailed investigation including hemogram, liver, and kidney-function tests, to exclude other diseases that may cause sleep disturbances. Sleep quality was assessed by using the Chinese version of the PSQI; depressive symptoms, with Zung self-rating depression scale (SDS), and anxiety symptoms with the Zung self-rating anxiety scale (SAS). Overall assessment of health score in AS patients was determined from a visual analogue scale (VAS), recording from 0 cm (none) to 10 cm (very severe); the pain score by visual analogue scale (VAS, 0 to 10 cm), included nocturnal pain (last week/spine/at night due to AS) and total back pain (last week/spine/due to AS). The disease activity was evaluated by the BASDAI, CRP, ESR, and duration of morning stiffness; the functional state was assessed by BASFI, metrologic measurements by fingertip-to-floor distance, and BASMI. The PSQI, SDS, and SAS were used in healthy controls to assess sleep, depression, and anxiety respectively.

### Sleep quality

Sleep during the month preceding the evaluation was examined by using the PSQI, which includes 19 questions completed by the subject. These 19 items were broken down into the following seven components: subjective sleep quality, sleep latency, sleep duration, habitual sleep efficiency, sleep disorders, use of hypnotics, and daytime dysfunction. Sleep latency is assessed by two questions rated according to time to fall asleep. Sleep duration by one question rated 4 on a Likert scale from >7 to <5 hours, and sleep efficiency by hours asleep divided by total of hours in bed. Use of hypnotics and poor daytime functioning are rated by a 4-point Likert scale (not during the past month, less than once per week, once or twice per week, 3 or more times per week). Sleep disorders were assessed with nine questions focused on waking up in the middle of the night or early in the morning, getting up to go to the toilet, difficulty breathing properly, coughing or snoring loudly, being too cold, being too hot, having nightmares, experiencing pain, or other reasons for disturbed sleep. Each question is rated with a 4-point Likert scale (not during the past month, less than once per week, once or twice per week, 3 or more times per week). Subjective sleep is evaluated with one question rated by a 4-point Likert scale from very good to very bad. The seven components were each scored from 0 (no difficulty) to 3 (severe difficulty), and summed, to give an overall score ranging from 0 to 21. Participants were dichotomized into a poor-sleep group if the PSQI was >7.0 and a good-sleep group if the PSQI was ≤7.0 [22].

### Depressive symptoms

The depression levels of the patients were evaluated by using the SDS. This scale consists of 20 questions that examine the somatic, affective, and psychological symptoms associated with depression. Each question is scored between 1 and 4 (rarely, sometimes, frequently, and always). The total score varies between 20 and 80. Higher scores reflected more-severe depression. A score of 50 to 69 shows the existence of depression, and a score of 70 or higher means extreme depression [23].

### Anxiety symptoms

The revised SAS was used to evaluate the level of anxiety-related symptoms during the last week before the survey. This self-administered test has 20 questions, with 15 items worded toward increasing anxiety levels and five questions worded toward decreasing anxiety levels. Each question was scored on a scale of 1 to 4 (rarely, sometimes, frequently, and always). The scores ranged between 20 and 80. A score of 50 to 69 shows the existence of anxiety, and a score of 70 or higher means extreme anxiety [24].

### Data analysis

Descriptive statistics were used for assessing the parameters related to disease. The differences in terms of variables that are studied in patients who are grouped as experiencing poor sleep and good sleep, according to the PSQI, were evaluated with independent sample Mann-Whitney *U *test and a *t *test. The relations between the sleep and the other evaluation parameters were examined with Spearman rank correlation analysis. Hierarchic multiple regression analysis was chosen to analyze the contribution of demographic, medical, and psychological variables to sleep disturbances. Stepwise multiple regression analysis was designed to find the most powerful predictor of sleep disturbances. Statistical analysis was performed by using SPSS 17.0, and the level of significance was set at *P *< 0.05.

## Results

### Patient characteristics

Of the 318 AS patients who agreed to participate in this study, 314 (98.7%) returned their completed questionnaires, and four patients were no longer interested and/or had no time to participate.

Demographic and clinical characteristics of the patients are given in Table 1. The 314 consecutive patients with AS (234 men, 80 women) were included in the study. The mean age of patients was 27.6 ± 8.3 years, and the mean disease duration was 6.1 ± 4.9 years. The mean PSQI, SDS, and SAS scores of patients were 6.6 ± 3.6, 47.3 ± 10.5, and 48.4 ± 8.1, respectively.

**Table 1:** Demographic and disease-related characteristics of patients with ankylosing spondylitis

Variables	Values
Age (years)^a^	27.65 ± 8.34
Gender: female, *n *(%)^b^	80 (25.5)
Years of education, *n *(%)^b^	
≤6 years	8 (2.5)
7-9 years	54 (17.2)
10-12 years	78 (24.8)
13-17 years	156 (49.7)
≥18 years	8 (5.7)
Number employed, *n *(%)^b^	191 (60.8)
Number of students, *n *(%)^b^	89 (28.3)
Married, *n *(%)^b^	177 (56.4)
ESR (mm/h)^a^	16.80 ± 18.78
CRP (mg/dl)^a^	1.62 ± 3.45
Disease duration (years)^a^	6.07 ± 4.90
Morning stiffness (minutes)^a^	11.90 ± 27.80
Fingertip-to-floor distance (cm)^a^	9.77 ± 13.70
Overall assessment of health (VAS)^a^	5.34 ± 2.87
Nocturnal pain (VAS)^a^	3.69 ± 2.95
Total back pain (VAS)^a^	3.85 ± 2.70
BASMI^a^	1.39 ± 1.89
BASDAI (VAS)^a^	3.58 ± 2.09
BASFI (VAS)^a^	1.64 ± 2.17
PSQI^a^	6.62 ± 3.62
SDS^a^	47.26 ± 10.45
SAS^a^	48.36 ± 8.10

**^a^**Mean ± SD. **^b^**Percentage. *N *= 314. BASDAI, Bath Ankylosing Spondylitis Disease Activity Index; BASFI, Bath Ankylosing Spondylitis Functional Index; BASMI, Bath Ankylosing Spondylitis Metrology Index; CRP, C-reactive protein; ESR, erythrocyte sedimentation rate; PSQI, Pittsburgh Sleep Quality Index; SAS, Self-rating Anxiety Scale; SDS, Self-rating Depression Scale; VAS, visual analogue scale.

### Sleep-disturbances parameters

As shown in Table 2, components of PSQI include subjective sleep quality, sleep latency, sleep duration, habitual sleep efficiency, sleep disorders, use of hypnotics, and daytime dysfunction. The seven components were each scored from 0 (no difficulty) to 3 (severe difficulty). When the score of the components was ≥2, the component was regarded as indicating sleep disturbance. We found that poor subjective sleep quality was 22.3%, sleep latency was 22.9%, sleep duration was 13.4%, habitual sleep efficiency was 25.6%, sleep disorders was 30%, use of hypnotics was 2.9%, and daytime dysfunction was 57.6% in patients with AS. The most prominent sleep disturbance was daytime dysfunction. Despite the high incidence of sleep disturbance in AS patients, they rarely use hypnotics.

**Table 2:** Descriptive statistics on the components of PSQI in cases

PSQI components score	0	1	2	3
Subjective sleep quality, *n *(%)	78 (24.8)	166 (52.9)	58 (18.5)	12 (3.8)
Sleep latency, *n *(%)	103 (32.8)	139 (44.3)	46 (14.6)	26 (8.3)
Sleep duration, *n *(%)	155 (49.4)	117 (37.3)	31 (9.9)	11 (3.5)
Habitual sleep efficiency, *n *(%)	136 (43.3)	98 (31.2)	41 (13.1)	39 (12.4)
Sleep disorders, *n *(%)	39 (12.4)	181 (57.6)	84 (26.8)	10 (3.2)
Use of sleep medications, *n *(%)	297 (94.6)	8 (2.5)	4 (1.3)	5 (1.6)
Daytime dysfunction, *n *(%)	35 (11.1)	98 (31.2)	109 (34.7)	72 (22.9)

*N ***= 314**.

### Mann-Whitney *U *test and *t *test

The prevalence of poor sleep was 35.4%, and the mean total PSQI score was 6.62 ± 3.62 in participants, compared with 22.9% and 5.50 ± 2.51, respectively, in the control group. The total PSQI score of patients with AS was significantly higher than that of the control participants (6.62 ± 3.62 versus 5.50 ± 2.51; *P *= 0.020), and four of the seven components of PSQI in AS patients were significantly different from controls: sleep latency, sleep duration, habitual sleep efficiency, and sleep disorders (see Table 3).

**Table 3:** Comparison of the components of PSQI in cases and controls

PSQI components score	Cases *n *= 314	Controls *n *= 102	*P *value
Subjective sleep quality	1.01 ± 0.77	0.89 ± 0.79	0.127
Sleep latency	0.98 ± 0.89	0.69 ± 0.88	0.001
Sleep duration	0.68 ± 0.79	1.29 ± 0.86	0.000
Habitual sleep efficiency	0.95 ± 1.03	0.25 ± 0.64	0.000
Sleep disorders	1.21 ± 0.69	0.74 ± 0.63	0.000
Use of sleep medications	0.10 ± 0.46	0.01 ± 0.09	0.055
Daytime dysfunction	1.69 ± 0.95	1.63 ± 1.00	0.635
Total	6.62 ± 3.62	5.50 ± 2.51	0.020

*P *values were obtained with the Mann-Whitney *U *test. *PSQI*, Pittsburgh Sleep Quality Index.

The Mann-Whitney *U *test and a *t *test were performed between patients who were good and poor sleepers (see Table 4). Poor-sleep patients with had significantly higher levels of morning stiffness, BASFI, overall poor assessment of health, nocturnal pain, total back pain, BASDAI, ESR, CRP, SDS, and SAS (*P *< 0.05) than did good-sleep patients. With regard to age, years of education, disease duration, fingertip-to-floor distance, and BASMI score, no statistically significant differences were found between the two groups (*P *> 0.05)

**Table 4:** Comparison between poor and good sleepers (sorted by *P *value)

Variables	PSQI ≤7	PSQI >7	*P*
	*n *= 203	*n *= 111	
SDS	44.15 ± 9.44	52.95 ± 9.83	0.000
SAS	45.78 ± 7.07	53.07 ± 7.77	0.000
BASDAI	2.98 ± 1.87	4.66 ± 2.03	0.000
BASFI	1.23 ± 1.83	2.39 ± 2.51	0.000
Nocturnal pain (VAS)	2.89 ± 2.64	5.17 ± 2.94	0.000
Total back pain (VAS)	3.12 ± 2.49	5.18 ± 2.57	0.000
Overall assessment of health (VAS)	4.67 ± 2.79	6.59 ± 2.58	0.000
Morning stiffness (minutes)	9.53 ± 26.48	16.24 ± 29.70	0.002
Age (years)	26.83 ± 8.03	29.70 ± 8.59	0.002
ESR (mm/h)	14.51 ± 15.44	20.98 ± 23.21	0.018
CRP (mg/dl)	1.32 ± 1.65	2.16 ± 5.33	0.039
Years of education	4.46 ± 0.89	4.25 ± 0.98	0.058
BASMI	1.29 ± 1.87	1.59 ± 1.93	0.069
Disease duration (years)	5.70 ± 4.57	6.74 ± 5.40	0.116
Fingertip-to-floor distance (cm)	9.65 ± 13.51	9.99 ± 14.09	0.859

BASDAI, Bath Ankylosing Spondylitis Disease Activity Index; BASFI, Bath Ankylosing Spondylitis Functional Index; BASMI, Bath Ankylosing Spondylitis Metrology Index; CRP, C-reactive protein; ESR, erythrocyte sedimentation rate; PSQI, Pittsburgh Sleep Quality Index; SAS, Self-rating Anxiety Scale; SDS, Self-rating Depression Scale; VAS, visual analogue scale.

### Bivariate associations

As shown in Table 5, Spearman rank correlation coefficients were computed to identify correlates of components of PSQI, demographic, clinical, and psychogenic variables in our sample of AS patients. PSQI and almost all components of the PSQI score were associated with age, years of education, ESR, CRP, morning stiffness, overall assessment of health, nocturnal pain, total back pain. The BASDAI, BASFI, SDS, and SAS (*P *< 0.001), were not associated with disease duration, fingertip-to-floor distance, and BASMI (*P *> 0.05). In these, sleep scores was moderately related to depression scores (rho, 0.467), nocturnal pain (rho, 0.487), total back pain (rho, 0.485), BASDAI (rho, 0.479), overall assessment of health (rho, 0.386), and BASFI (rho, 0.312), and highly related to anxiety scores (rho, 0.522).

**Table 5:** Correlation coefficients between components of PSQI, demographic, clinical, and psychogenic variables (Spearman rho)

Variables	PSQI	Subjective sleep quality	Sleep latency	Sleep duration	Habitual sleep efficiency	Sleep disorders	Use of sleep medications	Daytime dysfunction
Age (years)	0.165**	0.073	0.142*	0.074	0.065	0.193**	0.135*	0.139*
Years of education	-0.149**	0.003	-0.206**	-0.083	-0.119*	-0.047	-0.186**	-0.099
ESR (mm/h)	0.163**	0.187**	0.086	0.070	0.171**	0.102	0.062	0.044
CRP (mg/dl)	0.164**	0.172**	0.072	0.034	0.159**	0.134*	0.040	0.125*
Disease duration (years)	0.094	0.051	0.091	0.011	-0.013	0.095	-0.014	0.133*
Morning stiffness (minutes)	0.250**	0.219**	0.185**	0.103	0.177**	0.173**	0.069	0.180**
Fingertip-to-floor distance (cm)	0.04	0.040	0.010	-0.038	-0.025	0.084	0.049	0.114*
Overall assessment of health (VAS)	0.386**	0.258**	0.241**	0.073	0.200**	0.359**	0.174**	0.375**
Nocturnal pain (VAS)	0.487**	0.324**	0.337**	0.181**	0.330**	0.364**	0.129*	0.388**
Total back pain (VAS)	0.485**	0.319**	0.297**	0.199**	0.295**	0.386**	0.176**	0.424**
BASMI	0.093	0.044	0.057	0.023	0.105	0.122*	0.025	0.065
BASDAI	0.479**	0.322**	0.327**	0.130**	0.214**	0.421**	0.206**	0.467**
BASFI	0.312**	0.198**	0.168**	0.052	0.119*	0.421**	0.128*	0.313**
SDS	0.467**	0.290**	0.346**	0.124*	0.178**	0.438**	0.167**	0.448**
SAS	0.522**	0.347**	0.384**	0.173**	0.236**	0.493**	0.169**	0.439**

*P < 0.05; **P < 0.01. Spearman rho, <0.3, the weak correlation; Spearman rho, 0.3 to 0.5, the medium correlation; Spearman rho, >0.5, the high correlation. BASDAI, Bath Ankylosing Spondylitis Disease Activity Index; BASFI, Bath Ankylosing Spondylitis Functional Index; BASMI, Bath Ankylosing Spondylitis Metrology Index; CRP, C-reactive protein; ESR, erythrocyte sedimentation rate; PSQI, Pittsburgh Sleep Quality Index; SAS, Self-rating Anxiety Scale; SDS, Self-rating Depression Scale; VAS, visual analogue scale.

#### Hierarchic multiple regression analysis

The results of the hierarchic multiple regression analyses are shown in Table 6.

**Table 6:** Hierarchic multiple regression analysis of demographic, medical, and psychological variables in relation to PSQI

	Model 1		Model 2		Model 3		Model 4	
				
	B	*P *value	B	*P *value	B	*P *value	B	*P *value
Age (years)	0.057	0.122	0.064	0.081	0.032	0.314	0.055	0.103
Gender (male)	0.738	0.108	0.098	0.823	-0.228	0.575	-0.435	0.292
Years of education	-0.583	0.011	-0.333	0.105	-0.266	0.181	-0.190	0.318
Student (yes)	-1.261	0.083	0.587	0.365	-0.805	0.203	-0.455	0.449
Employment (yes)	-1.068	0.078	-1.099	0.043	-0.638	0.225	-0.798	0.112
Married (yes)	0.174	0.788	0.168	0.769	0.010	0.985	0.011	0.984

ESR			0.007	0.535			0.009	0.399
CRP			-0.005	0.941			-0.022	0.706
Disease duration			-0.055	0.230			-0.052	0.214
Morning stiffness (minute)			0.001	0.919			0.001	0.904
Fingertip-to-floor distance (cm)			-0.011	0.481			-0.009	0.531
Overall assessment of health (VAS)			0.029	0.760			-0.039	0.658
Nocturnal pain (VAS)			0.267	0.012			0.258	0.009
Total back pain (VAS)			0.133	0.302			0.168	0.159
BASMI			-0.222	0.100			-0.149	0.233
BASDAI (VAS)			0.456	0.005			0.210	0.168
BASFI (VAS)			-0.067	0.595			-0.157	0.184

SDS					0.064	0.004	0.059	0.008
SAS					0.170	0.000	0.120	0.000

*R^2 ^*(%)	8.5		32.0		32.4		42.6	
Adjusted *R^2 ^*(%)	6.7		28.0		30.6		38.9	

Model 1 *F *= 4.756; *P *= 0.000; Model 2 *F *= 8.176; *P *= 0.000; Model 3 *F *= 18.290; *P *= 0.000; Model 4 *F *= 11.507; *P *= 0.000. BASDAI, Bath Ankylosing Spondylitis Disease Activity Index; BASFI, Bath Ankylosing Spondylitis Functional Index; BASMI, Bath Ankylosing Spondylitis Metrology Index; CRP, C-reactive protein; ESR, erythrocyte sedimentation rate; PSQI, Pittsburgh Sleep Quality Index; SAS, Self-rating Anxiety Scale; SDS, Self-rating Depression Scale; VAS, visual analogue scale.

Model 1 (demographic model) tests the contribution of demographic variables to sleep disturbances.

Model 2 (medical variables model) tests the contributions of medical and physiological variables to sleep disturbances after controlling for the demographic variables.

Model 3 (psychological model) describes the extent to which psychological variables contribute to sleep disturbances after controlling for the demographic variables. Models 1 through 3 are the results of hierarchic regression analyses, which are usually done in research to determine the importance of predictor variables once other predictor variables have already been entered into the equation.

Model 4 is the standard regression analysis in which all of the variables were entered simultaneously into the model to assess the relative contributions of these variables to sleep disturbances. This model takes into account the interrelations between predictor variables as well as the effects of predictor variables on the outcome variable (sleep disturbances). The first model testing the contributions of demographic variables to sleep disturbances was found to be statistically significant (*R^2 ^*= 0.85). In this model, years of education (*P *= 0.011) contributed significantly to sleep disturbances. The addition of medical variables (model 2) resulted in a significant increase in the *R^2 ^*value (*R^2 ^*= 0.32). In this model, Nocturnal pain (*P *= 0.012) and BASDAI (*P *= 0.005) contributed significantly to sleep disturbances. Inclusion of psychological variables also significantly added to the demographic set (*R^2 ^*= 0.324). In this model, SDS (*P *= 0.004) and SAS (*P *= 0.000) contributed significantly to sleep disturbances. The medical and psychological variables explained an additional 23.9% of the variance in sleep disturbances. In the final full model (standard multiple regression analysis), in which all of the variables were entered simultaneously, only nocturnal pain (*P *= 0.009), SDS (*P *= 0.008), and SAS scores (*P *= 0.000) remained as significant determinants of sleep disturbances. The full model could explain 42.6% of the total variance in sleep disturbances.

#### Multiple stepwise regression analysis

The results of the stepwise multiple regression analyses are shown in Table 7. The SDS score (B = 0.059; *P *= 0.004), SAS score (B = 0.128; *P *= 0.000), nocturnal pain (B = 0.235; *P *= 0.013), and total back pain (B = 0.210; *P *= 0.044) were found to be significantly associated with sleep disturbances. The *R^2 ^*= 39.5 indicated that 39.5% variation of sleep disturbances could be explained by depression, nocturnal pain, and total back pain. None of the factors significantly influenced sleep disturbances (age, gender, years of education, occupation, marital status, ESR, CRP, disease duration, morning stiffness, fingertip-to-floor distance, BASMI, BASDAI, or BASFI). The stepwise multiple regression analyses revealed that anxiety was the maximal statistical contribution in predicting back pain (standardized coefficients of 0.287).

**Table 7:** Stepwise multiple regression analysis of demographic, physiological, medical, and psychological variables in relation to PSQI

	Independent	B	Standardized coefficients	*t*	*P*	*R^2^*(%)
Predictors						39.5
	SDS	0.059	0.170	2.884	0.004	
	SAS	0.128	0.287	4.759	0.000	
	Nocturnal pain (VAS)	0.235	0.191	2.511	0.013	
	Total back pain (VAS)	0.210	0.157	2.027	0.044	

PSQI, Pittsburgh Sleep Quality Index; SAS, Self-rating Anxiety Scale; SDS, Self-rating Depression Scale, VAS, visual analogue scale.

## Discussion

The current study is, to our knowledge, the largest to evaluate sleep disorders in AS patients. The relation between sleep disturbances and physical and psychological problems were described by the following six aspects.

### Characteristics of sleep in AS patients

Significant sleep disturbance was much more common in AS patients than in the control group. PSQI is a standard index for assessing sleep quality and is widely used in both clinical settings and public health practice. The prevalence of poor sleep was 35.4%, and the mean total PSQI score was 6.62 ± 3.62 in participants, compared with 22.9% and 5.50 ± 2.51, respectively, in the control group. This prevalence was lower than those reported, 54.1% and 64.5% of sleep disturbance, respectively, in other studies of AS patients [20,25].

Among seven components of PSQI, the most prominent component of sleep disturbances was daytime dysfunction. In total, 57.6% of participants reported various degrees of daytime dysfunction, including excessive daytime sleepiness, fatigue, and changes in mood. In another study in patients with AS [20], 66.4% experienced severe fatigue, and a positive relation was established between fatigue and sleep problems. These changes are reflected in patient reports of difficulty in concentrating, increased forgetfulness, an inability to make decisions, and falling asleep at the wheel of a motor vehicle. Those with moderate to severe cases of the disorder are at a higher risk of having a motor vehicle accident and may also have difficulties at work or school [26].

Another prominent component of PSQI was use of sleep medication. Despite the high incidence of sleep disturbance in AS patients, they rarely use hypnotics. The main reasons for impairment of chronic sleep disturbance were not paid attention to by patients, such as daytime sleepiness, fatigue, negative mood, and recognition [8-13]. Moreover, patients worry that long-term use of sleep medication could increase the risks of development of tolerance and dependence. Intervention for and prevention of sleep disturbances are becoming increasingly important. Recent studies [27,28] concluded that nonpharmacologic interventions are clinically effective for sleep disturbance, including sleep-hygiene education, bright-light therapy, and cognitive and behavioral therapies.

### Sleep and pain

Pain is one of the most common symptoms of AS. However, it is a chicken-and-egg problem to determine the direction of causation between pain and sleep disturbance. Some studies [29-31] demonstrated an apparent bidirectionality in the relation between pain causing sleep dysfunction and sleep dysfunction aggravating pain. This cross-sectional study demonstrates a strong association between not only sleep disturbance and pain (nocturnal pain, total back pain), but also the components of PSQI (subjective sleep quality, sleep latency, sleep duration, habitual sleep efficiency, sleep disorders, use of sleep medications, daytime dysfunction) and pain in AS patients.

These findings are consistent with a previous study [32] showing an association between sleep disturbance and clinical pain severity among RA patients. The mechanism of interaction between sleep disturbance and pain, so far not determined, could be understood with two factors. First, from the perspective of neurobiology, sleep disruption, fragmentation, or restriction produces hyperalgesia and can interfere with analgesic treatments involving opioidergic and serotonergic mechanisms of action [33-36]. Second, from the perspective of psychology, sleep disturbance lowers the pain threshold and amplifies the pain signals, resulting in increased attention to pain, and more negative pain-focused emotions and cognitions [3]. Thus, patients experience increased pain by hyperalgesia, reduced activity levels, and further disrupted sleep in a positive-feedback-loop pattern.

### Sleep and disease activity

A close relation exists between sleep quality and disease activity in AS patients. In the current study, analyses were performed between patients who were good and poor sleepers; poor-sleep patients had significantly higher levels of morning stiffness, BASDAI, ESR, and CRP compared with good sleepers (*P *< 0.05). These findings indicate that sleep quality of AS patients is closely related to disease activity. CRP could directly and/or indirectly affect sleep quality. Some evidence [37-40] stems from studies that indicate that poor sleep is associated with inflammatory responses, indicated by increased CRP. Okun *et al*. [40] evaluated cross-sectional relations between self-reported sleep and three inflammatory markers (interleukin-6, tumor necrosis factor-α, and CRP) in community-dwelling nonpregnant women. Findings showed that poor sleep continuity and quality were related to high levels of CRP. Furthermore, Lee *et al*. [2] examining the association between disease activity, sleep, and pain sensitivity in rheumatoid arthritis, found that CRP was strongly associated with the pain threshold. Speculating from this result, we suspect that the CRP could indirectly affect sleep quality via a decreased pain threshold.

Tumor necrosis factor (TNF) plays an important role in AS and sleep regulation. Several observations [41,42] strongly implicated TNF in the pathogenesis of AS, and elevated serum levels of TNF correlate with disease activity [43]. TNF-inhibitor therapies, such as thalidomide [44], etanercept [45], and infliximab [46], have been demonstrated to be effective in the treatment of AS. Moreover, TNF-α has been determined to be an important sleep-promoting substance [47,48]. Chennaoui *et al*. [47] reported that total sleep deprivation in young healthy men is associated with significant sleepiness, decrements in psychomotor performance, and increased secretion of the proinflammatory cytokine TNF-α. The similar results of sleep deprivation led to elevated levels of TNF-α had been obtained in another experiment [48]. In turn, many of the symptoms induced by sleep loss (for example, sleepiness, fatigue, poor cognition, and enhanced sensitivity to pain) can be elicited by injection of exogenous IL-1 or TNF [49]. Further study was done recently by Rudwaleit *et al*. [50], and the results indicated that the TNF inhibitor, adalimumab, improved sleep and sleep quality in patients with active AS.

### Sleep and depression

Sleep problems were the chief complaints of depression and anxiety; early awakening is the main disturbance of a depression disorder; and difficulty falling asleep is present in anxiety disorder. In the present study, analyses were performed between patients who were good and poor sleepers. Poor-sleep patients had significantly higher levels of SDS scores compared with good sleepers.

Sleep disturbances in relation to a depressed mood have been examined in the general population. Sayar *et al*. [51] reported that pain intensity, anxiety, and depression correlated significantly with poorer sleep quality. At the multivariate level, depression was found to be the only significant factor correlating with the quality of sleep, and the model explained 34% of the variance. In 2005, Costa *et al*. [52] compared systemic lupus erythematosus patients with healthy controls and insomniacs and concluded that only depressed mood was a significant independent determinant of global sleep quality.

The relation between sleep complaints and depressed mood is consistent with findings from another study of SLE [4]. Hultgren *et al*. [19] reported that depression was independently associated with three of the sleep parameters (quality, duration, and efficiency), whereas perceived stress contributed to global sleep quality. In this study, the association between depression and sleep disturbances was evident and significant on bivariate analysis, but did not remain significant on logistic regression analyses.

### Sleep and anxiety

To our knowledge, this is the first study to examine anxiety correlates of sleep problems in AS. Poor-sleep patients had significantly higher levels of SAS scores compared with those of good sleepers. Bivariate analyses indicated a significant relation between anxiety and sleep disturbances. The relation of sleep and anxiety, rarely has been studied separately, apparently less than that of sleep and depression. A number of studies [53-55] indicated that sleep problems usually were associated with depression and anxiety. However, anxiety was divided into trait-anxiety and state-anxiety in a study by Augner [56], who reported that subjective sleep quality was strongly negatively correlated with trait-anxiety, which is ingrained in a person's personality. Further study might indicate whether state-anxiety, characterized as a temporary change in a person's emotional state due to an outside factor, is correlated with sleep quality.

### Statistical contribution in predicting sleep disturbances

A number of studies [13,57-60] indicated that poor sleep or insomnia or both are risk factors for major depression. Gregory *et al*. [57] reported that sleep problems predict symptoms of depression at 10 years. Riemann *et al*. [59] also reported that insomnia symptoms for a period of more than 2 weeks predict an increased risk for developing depression within the following 3 years. A few studies [13,60,61], usually included in surveys of sleep and depression, found that poor sleep or insomnia could predict anxiety. In turn, little research exists on the role of depression and/or anxiety in sleep disturbances. In our hierarchic multiple regression analysis, the medical and psychological variables contributed significantly to the variance in sleep-disturbances scores, adding an additional 23.9% to the overall *R^2 ^*beyond that accounted for by demographic variables (*R^2^*, 8.5%), resulting in a final *R^2^*of 42.6%. Multiple stepwise regression analysis revealed that anxiety was the maximal statistical contribution in predicting sleep disturbances. It is worth mentioning that Jansson and Lindblom [11] conducted a population-based study providing prospective evidence that individuals liable to anxiety may be at particularly high risk of postevent sleep disturbances, at least during the first months after the event.

## Conclusions

In summary, half of the patients with AS are reported to have sleep disturbances, and multidimensional assessments have provided better understanding for the specific aspects of sleep. The factors that contributed to poor sleep in the study were overall assessment of health, pain, disease activity, BASFI, depression, and anxiety, even though it was observed that anxiety was the maximal statistical contribution in predicting sleep disturbances. Sleep dysfunction is importantly related to AS physical and psychological symptoms and deserves more attention in both research and clinical practice. Results highlight the high co-occurrence of sleep and AS problems, emphasizing the need for assessment and intervention of sleep disturbance in this population.

## Abbreviations

AS: ankylosing spondylitis; BASDAI: Bath Ankylosing Spondylitis Disease Activity Index; BASFI: Bath Ankylosing Spondylitis Functional Index; BASMI: Bath Ankylosing Spondylitis Metrology Index; CRP: C-reactive protein; ESR: erythrocyte sedimentation rate; PSQI: Pittsburgh sleep quality index; SAS: self-rating anxiety scale; SDS: self-rating depression scale; TNF: tumor necrosis factor.

## Competing interests

The authors declare that they have no competing interests.

## Authors' contributions

YL, SZ, and FH have contributed substantially in the processes of study design, data acquisition, and analysis and interpretation of data. XD contributed to the statistical analyses of the data. JZ has been involved in revising the manuscript critically for its important intellectual concept and also gave the final approval for its publication. All authors read and approved the final manuscript.
